# Plasma YKL-40 in Patients with Metastatic Colorectal Cancer Treated with First Line Oxaliplatin-Based Regimen with or without Cetuximab: RESULTS from the NORDIC VII Study

**DOI:** 10.1371/journal.pone.0087746

**Published:** 2014-02-03

**Authors:** Line S. Tarpgaard, Tormod K. Guren, Bengt Glimelius, Ib J. Christensen, Per Pfeiffer, Elin H. Kure, Halfdan Sorbye, Tone Ikdahl, Mette Yilmaz, Julia S. Johansen, Kjell Magne Tveit

**Affiliations:** 1 Department of Oncology, Odense University Hospital, Odense, Denmark and University of Southern Denmark, Odense, Denmark; 2 Department of Oncology, Oslo University Hospital, Oslo, Norway; 3 Departments of Radiology, Oncology and Radiation Science, Uppsala University, Uppsala, Sweden and Department of Oncology and Pathology, Karolinska Institute, Stockholm, Sweden; 4 The Finsen Laboratory, Copenhagen University Hospital, Copenhagen, Denmark and Biotech Research and Innovation Center (BRIC), University of Copenhagen, Copenhagen, Denmark; 5 Department of Genetics, Institute for Cancer Research, Oslo University Hospital, Oslo, Norway; 6 Department of Oncology, Haukeland University Hospital, Bergen, Norway; 7 Department of Oncology, Aalborg Hospital, Aalborg, Denmark; 8 Departments of Oncology and Medicine, Herlev Hospital, Copenhagen University Hospital, Copenhagen, Denmark; Sudbury Regional Hospital, Canada

## Abstract

**Background:**

We aim to test the hypothesis that high plasma YKL-40 is associated with short progression-free survival (PFS) and overall survival (OS) in patients with metastatic colorectal cancer (mCRC) treated with first-line oxaliplatin and 5-flourouracil with or without cetuximab.

**Patients and Methods:**

A total of 566 patients in the NORDIC VII Study were randomized 1∶1∶1 to arm A (Nordic FLOX), arm B (Nordic FLOX + cetuximab), or arm C (Nordic FLOX + cetuximab for 16 weeks followed by cetuximab alone as maintenance therapy). Pretreatment plasma samples were available from 510 patients. Plasma YKL-40 was determined by ELISA and dichotomized according to the age-corrected 95% YKL-40 level in 3130 healthy subjects.

**Results:**

Pretreatment plasma YKL-40 was elevated in 204 patients (40%), and median YKL-40 was higher in patients with mCRC than in healthy subjects (age adjusted, *P*<0.001). Patients with elevated YKL-40 had shorter PFS than patients with normal YKL-40 (7.5 vs. 8.2 months; hazard ratio (HR)  = 1.27 95% confidence interval (CI) 1.05–1.53 *P* = 0.013) and shorter OS (16.8 vs. 23.9 months; HR = 1.33, 1.04–1.69, *P* = 0.024). Multivariate Cox analysis demonstrated that elevated pretreatment YKL-40 was an independent biomarker of short OS (HR = 1.12, 1.01–1.25, *P* = 0.033). The ratio of the updated plasma YKL-40 (i.e. level after 1, 2, 8 weeks of treatment, and at end of treatment compared to the baseline level) was associated with OS (HR = 1.27, 1.06–1.52, *P* = 0.011).

**Conclusions:**

Plasma YKL-40 is an independent prognostic biomarker in patients with mCRC treated with first-line oxaliplatin-based therapy alone or combined with cetuximab.

## Introduction

Research into biological markers hopes to provide the clinician with an opportunity to choose the best treatment for the individual patient. Several clinical and laboratory values give prognostic information regarding treatment strategy for patients with metastatic colorectal cancer (mCRC). *KRAS* status is presently the only biomarker routinely used to select patients with mCRC for epidermal growth factor receptor (EGFR) inhibition-targeted therapy. Patients with wild type (wt) KRAS mCRC benefit from inhibition in combination with FOLFIRI or FOLFOX [1]–[3], even though the effect is not confirmed in all phase III studies, where EGFR-inhibitors were combined with some oxaliplatin-based regimes [4], [5]. In the NORDIC VII study, a survival benefit of adding cetuximab to the Nordic FLOX regimen could not be confirmed [5]. Identification of new predictive and prognostic biomarkers is essential.

YKL-40 (also named chitinase-3-like 1 protein) is a highly conserved glycoprotein [6], and its gene is located on chromosome 1q32.1 [7]. The YKL-40 protein is highly expressed in embryonic tissue characterized by rapid proliferation and differentitation [8]. In adults, high YKL-40 expression is observed in cells with high cellular activity [9]. YKL-40 is produced by cancer cells, macrophages, and neutrophils [6], [7], [10] and is stimulated by hypoxia [11] and IL-6 [12]. YKL-40 also induces cancer angiogenesis both independently and through stimulating vascular endothelial growth factor [13]–[16]. Furthermore YKL-40 up-regulates pro-inflammatory mediators [17] and activates the Akt signaling pathway in colonic epithelial cells [18]. Recently, it has beed demonstrated that YKL-40 regulates cellular and tissue responses via the IL-13 receptor α2 and it activates macrophage mitogen-activated protein kinase, protein kinase B/AKT, and Wnt/β-catenin signaling [19].

YKL-40 is known to be an independent prognostic biomarker of short overall survival (OS) in patients with different types of cancers [10] and in patients with CRC after surgery [20], [21]. Little is known about the prognostic value of YKL-40 in patients with mCRC [22]. Furthermore, high plasma YKL-40 in subjects from the general population is associated with an increased risk of developing gastrointestinal cancer [23] and death from gastrointestinal cancer [24], [25].

EGFR mediates stimulation of cellular proliferation, survival, and motility [26] and is involved in tumorigenesis if abnormally activated [27]-[29]. Alterations within the EGFR signaling cascade like gene mutations, gene amplifications, and protein over-expression play a role in colorectal carcinogenesis [30]. EGFR is an established target for cancer treatment, and inhibition of the receptor has shown clinical efficacy in patients with mCRC [31].

In the present study, we tested the hypothesis that an elevated plasma concentration of YKL-40 is associated with short PFS and OS in patients with mCRC treated with first-line Nordic FLOX given continuously or intermittently with or without cetuximab in the NORDIC VII Study. We also examined whether increases in plasma YKL-40 during treatment were associated with poor prognosis.

## Patients and Methods

### Study Design and Patients

All participating patients provided written informed consent, and the study (including biomarker analysis) was approved by the Regional Ethics Committee (VEK ref. 20050053). Further details about the study have been published [5].

**Figure 1:**
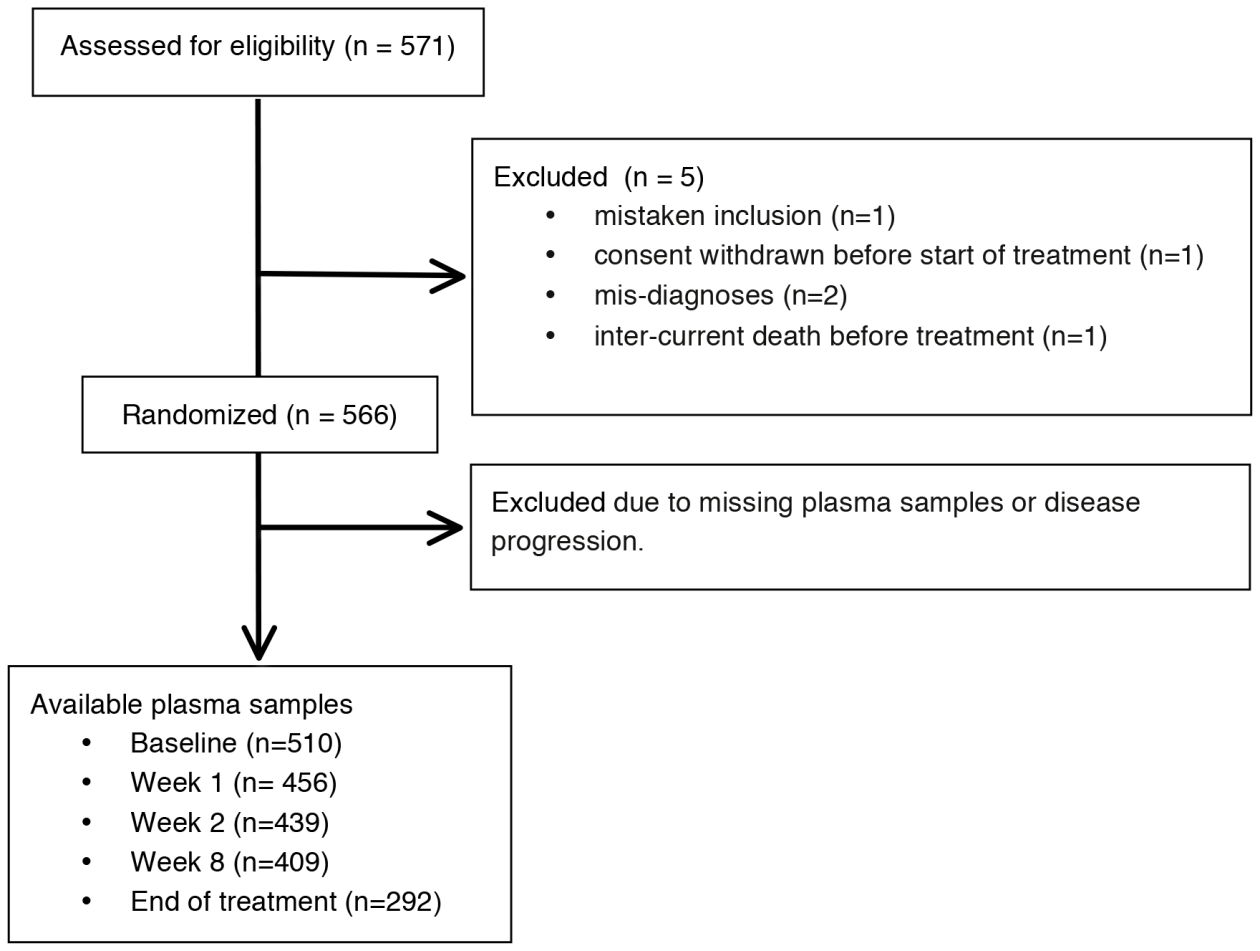
Consort diagram indicating sample sizes at each stage during the study.

The NORDIC VII Study (http://clinicaltrials.gov/show/NCT00145314) was an open-label randomized investigator-initiated, multicenter phase III trial [5], with a total of 571 patients enrolled from 32 Nordic centers. Five patients were later classified as not eligible (one mistaken inclusion; one consent withdrawn before start of treatment; two mis-diagnoses, with no metastatic or measurable disease; and one intercurrent death before treatment). The remaining 566 patients were randomly assigned in a 1∶1∶1 ratio to the three treatment arms: Nordic FLOX: 5-FU i.v. bolus 500 mg/m^2^ and FA 60 mg/m^2^ day 1–2, oxaliplatin 85 mg/m^2^ day 1 every two week until progression (arm A); Nordic FLOX plus cetuximab (400 mg/m^2^ day 1, then 250 mg/m^2^ weekly) until progression (arm B) and Nordic FLOX + cetuximab for 16 weeks, and weekly cetuximab as maintenance treatment until progression, followed by re-introduction of FLOX (arm C). The main inclusion criteria were histologically confirmed mCRC (adenocarcinoma); age >18 years and <75 years; WHO performance status (PS) ≤2; no prior chemotherapy for advanced or mCRC, non-resectable and measurable disease according to the Response Evaluation Criteria in Solid Tumors (RECIST version 1.0); adjuvant chemotherapy not given for at least 6 months; no previous oxaliplatin; adequate hematological, renal, and liver function; no peripheral neuropathy; and no other serious illness or medical conditions. The patients were treated until disease progression and followed until death or April 30, 2009. Pretreatment plasma samples were available from 510 (90%) patients, after 1 week of treatment from 456 patients, after 2 weeks from 439 patients, after 8 weeks from 409 patients, and at the end of treatment from 292 patients (Figure 1). In the NORDIC VII Study blood and tumor tissue were collected for subsequent explorative biomarker studies. Plasma YKL-40 was the first retrospective biomarker study on the blood samples.

### YKL-40 analysis

Plasma samples were collected and handled according to standard operating procedures. YKL-40 concentrations in EDTA plasma were determined in duplicate, in samples stored at minus 80°C, by a commercial enzyme-linked immunosorbent assay (Quidel, Santa Clara, CA, USA). The detection limit was 15 µg/L. The intra-assay coefficient of variation (CV) was <5% and the inter-assay CV was <6%. All samples from each patient were analyzed on the same plate to reduce inter-assay CV.

### Plasma YKL-40 in healthy subjects

The reference intervals for plasma YKL-40 were determined in 3130 healthy subjects (1837 women, 1293 men) aged 21 to 84 years from the Danish general population, the Copenhagen City Heart Study [32]. They had no known disease at the time of blood sampling in 1991–1994 and remained healthy and alive during the 16-year follow-up period [32]. The median plasma YKL-40 in these 3130 healthy subjects was 40 µg/L. The cut-off value for plasma YKL-40 was defined as higher or lower than the upper normal age-corrected level for the 95 percentile [32].

### Mutation analysis of *KRAS* and *BRAF*

Genomic DNA was extracted from formalin-fixed paraffin-embedded 10-µm tissue sections with 65% to 70% (median) tumor cells using QIAamp DNA Micro Kit (Qiagen, Venio, the Netherlands). Tumor DNA was screened for seven *KRAS* mutations in codons 12 (G12D, G12A, G12V, G12S, G12C, G12R) and 13 (G13D) using the TheraScreen *KRAS* kit (DxS, Manchester, United Kingdom) and for the *BRAF* V600E mutation [5].

### Statistical analysis

The primary endpoint for this biomarker study was OS, determined as the time from the baseline blood sample before start of treatment to time of death from all causes. Clinical information on disease status and OS was updated April 30, 2009. The median follow-up time was 37 months (24–53 months). Patients who were alive on this date were censored. The secondary endpoint was PFS (primary endpoint of the NORDIC VII Study). Descriptive statistics are presented by their median levels and ranges. Analyses of PFS and OS were done using Cox proportional hazards models. Plasma concentrations of YKL-40 were entered categorically as elevated vs. normal level (using the 95 percentile in healthy subjects (age-corrected) was used as cut-off) [32] or continuously as the actual value (log transformed) on the log scale (base 2). Missing values for serum CEA (*N* = 25), serum CRP (*N* = 21), serum alkaline phosphatase (*N* = 7), and *BRAF* and *KRAS* (*N* = 100) were categorized separately and included in the final multivariate analysis. Furthermore, we have taken into account that the tumor cannot be double mutated in terms of *BRAF* and *KRAS*. Analysis of response to treatment was perfomed using logistic regression, and the results are presented using odds ratios (OR) with 95% confidence limits (CI). Survival probabilities for OS were estimated by the Kaplan-Meier method and tests for differences between strata were done using log-rank statistics. Graphical presentation using Kaplan-Meier estimates of PFS and OS is shown by grouping patients according to elevated vs. normal plasma YKL-40 level. Model assessment was conducted using graphical methods, Schoenfeld and martingale residuals and internal cross validation. Analyses of updated plasma YKL-40 levels during treatment were performed using a Cox proportional hazard model with plasma YKL-40 as a time-dependent covariate. This model includes treatment (arm A, B, and C) and *KRAS* status. Kaplan-Meier estimates of survival probability using a landmark at 8 weeks of treatment were done for PFS and OS. P-values less than 5% were considered statistically significant. All calculations were performed using SAS (version 9.1, SAS Institute, Cary, NC, USA).

The results of this project are reported in accordance with the REMARK guidelines [33].

## Results

### Pretreatment plasma YKL-40 and demographic characteristics of the patients

The baseline demographic characteristics of the 510 patients with a pretreatment plasma YKL-40 measurement are shown in Table 1. The three study populations are demographically comparable and not different from the total intention-to-treat population of 566 patients with mCRC. Patients had significantly higher pretreatment plasma YKL-40 than healthy subjects (age adjusted, *P*<0.001). Plasma YKL-40 was higher than the upper normal level (age-corrected 95 percentile used as cut-off) in 40% of all patients. Plasma YKL-40 was not associated with *KRAS* (*P* = 0.41) or *BRAF* status (*P* = 0.88). Missing values of serum CEA, serum CRP, serum ALP, *KRAS* and *BRAF* were included as separate strata, complete case analysis (*N* = 378 patients included) of PFS and OS yielded almost the same estimates in multivariate analysis with broader CI's (data not shown).

**Table 1:** Demographic and Baseline Clinical Characteristics of the 510 patients with mCRC included in the Nordic VII Study with pretreatment plasma YKL-40.

Variable		*N* (%)	Median plasma YKL-40 in µg/L (range)	*P*-value for Wilcoxon test
Plasma YKL-40	Normal	306 (60)		
	Elevated	204 (40)		
Sex	Males	301 (59)	120 (23–1773)	0.36
	Females	209 (41)	121 (16–3945)	
WHO PS	0	342 (67)	104 (16–1773)	<0.0001
	1	146 (29)	151 (49–3945)	
	2	22 (4)	420 (46–1128)	
Location	Colon	302 (60)	129 (23–3945)	0.024
	Rectum	208 (40)	109 (16–1290)	
Number of metastatic sites	1	150 (29)	121 (16–3945)	0.41
	>1	360 (71)	120 (23–1773)	
Adjuvant chemotherapy	Yes	45 (9)	94 (23–240)	0.04
	No	465 (91)	124 (16–3945)	
*KRAS*	WT	273 (61)	119 (23–3945)	0.41
	Mutant	175 (39)	118 (23–1290)	
*BRAF*	WT	362 (89)	116 (23–1773)	0.88
	Mutant	47 (11)	124 (23–1494)	
Alkaline phosphatase	Normal	265 (52)	106 (16–1158)	<0.0001
	Elevated	245 (48)	225 (27–3945)	

### Pre-treatment plasma YKL-40, PFS, and OS

The median PFS and OS were 7.9 months (95% CI 7.5–8.3 months) and OS 20.3 months (95% CI 18.2–22.4 months), respectively. Univariate Cox analyses including all patients showed that elevated pretreatment plasma YKL-40 (dichotomized) was associated with short PFS compared to patients with normal plasma YKL-40 (7.5 months vs. 8.2 months; hazard ratio (HR)  = 1.27; 95% CI; 1.05–1.53; *P* = 0.013). This was found in patients treated with FLOX alone (arm A: HR = 1.42; 95% CI 1.02–1.98; *P* = 0.04), while there was no statistically significant difference in PFS among patients treated with FLOX and cetuximab (arm B: HR = 1.23; 95% CI 0.89–1.69; *P* = 0.20; and arm C: HR = 1.17; 95% CI 0.85–1.61; P = 0.50). There was no interaction between pre-treatment plasma YKL-40 and treatment arm (OS: *P* = 0.78, for treatment arm X *KRAS* mutational status X pre-treatment plasma YKL-40 *P* = 0.24; PFS: *P* = 0.30; for treatment arm X *KRAS* mutational status X pew-treatment plasma YKL-40, *P* = 0.47).

Univariate Cox analysis including all patients showed that elevated pretreatment plasma YKL-40 (dichotomized) was associated with short OS compared to patients with normal plasma YKL-40 (16.8 months vs. 23.9 months; HR = 1.33; 95% CI 1.04–1.69; *P* = 0.024) (data not shown). If plasma YKL-40 was included as a log transformed continuous variable, similar results were found for PFS (HR = 1.11; 95% CI 1.03–1.20; *P* = 0.006) and OS (HR = 1.18; 95% CI 1.06–1.32; *P* = 0.002) (Table 2).

**Table 2:** Univariate and multivariate Cox analyses of PFS and OS in 510 patients with mCRC included in the Nordic VII Study according to pretreatment plasma YKL-40 and clinical parameters.

	Progression-free Survival						Overall Survival					
	Univariate Cox analyses			Multivariate Cox analyses			Univariate Cox analyses			Multivariate Cox analyses		
	HR	95% CI	*P* value	HR	95% CI	*P* value	HR	95% CI	*P* value	HR	95% CI	*P* value
Treatment arm B vs A	0.95	0.76–1.29	0.71	0.95	0.75–1.19	0.66	1.07	0.82–1.39	o.062	1.02	0.78–1.33	0.98
Treatment arm C vs A	1.22	0.98–1.53	0.08	1.27	1.01–1.59	0.039	1.09	0.84–1.41	0.51	1.02	0.79–1.33	0.88
Plasma YKL-40 (log)*	1.11	1.03–1.20	0.006	1.00	0.91–1.09	0.97	1.18	1.06–1.32	0.002	1.17	1.05–1.30	0.88
Age per 10 years	0.94	0.85–1.04	0.21	0.93	0.84–1.03	0.17	0.98	0.87–1.11	0.79	0.99	0.88–1.12	0.85
Sex, Female vs. male	1.09	0.91–1.32	0.34	0.97	0.80–1.16	0.77	0.89	0.71–1.12	0.33	0.83	0.66–1.03	0.092
*KRAS,* Mutant vs. WT	1.16	0.95–1.42	0.15	1.37	1.09–1.71	0.006	1.06	0.84–1.34	0.63	1.50	1.13–1.91	0.004
*BRAF*, Mutant vs. WT	1.99	1.45–2.72	<0.0001	2.56	1.81–3.63	<0.0001	4.55	3.03–6.84	<0.0001	4.71	3.21–6.93	<0.0001
No. of metastatic sites, >1 vs. 1	1.42	1.16–1.75	0.0007	1.40	1.14–1.72	0.001	1.51	1.19–1.92	0.0008	1.44	1.13–1.85	0.003
WHO PS, ≥1 vs. 0	1.65	1.36–2.00	<0.0001	1.40	1.13–1.73	0.002	1.98	1.59–2.46	<0.0001	1.55	1.21–1.97	0.0003
Serum CRP, Elevated vs. normal	1.52	1.25–1.84	<0.0001	1.35	1.09–1.68	0.006	1.65	1.32–2.07	<0.0001	1.31	1.03–1.69	0.030
Serum CEA, Elevated vs. normal	1.64	1.27–2.10	<0.0001	1.62	1.24–2.12	0.0005	1.90	1.40–2.58	<0.0001	1.94	1.39–2.70	<0.0001

All treatment groups are combined.

HR =  Hazard ratio. CI =  Confidence interval. *Plasma YKL-40 was included as a log transformed continuous variable.

Multivariate Cox analysis (YKL-40, number of metastatic sites, WHO PS, *KRAS* and *BRAF* status, serum CRP, serum CEA, serum ALP, age, and sex) showed that elevated pretreatment plasma YKL-40 was an independent biomarker of short OS (continuous variable HR = 1.12; 95% CI 1.01–1.25; *P* = 0.033) (Table 2). However, this was not found if plasma YKL-40 was included in the multivariate analysis as a dichotomized variable. There was no association between plasma YKL-40 and PFS (continuous variable; HR = 0.99; 95% CI 0.90–1.08; *P* = 0.75) (Table 2).

**Figure 2:**
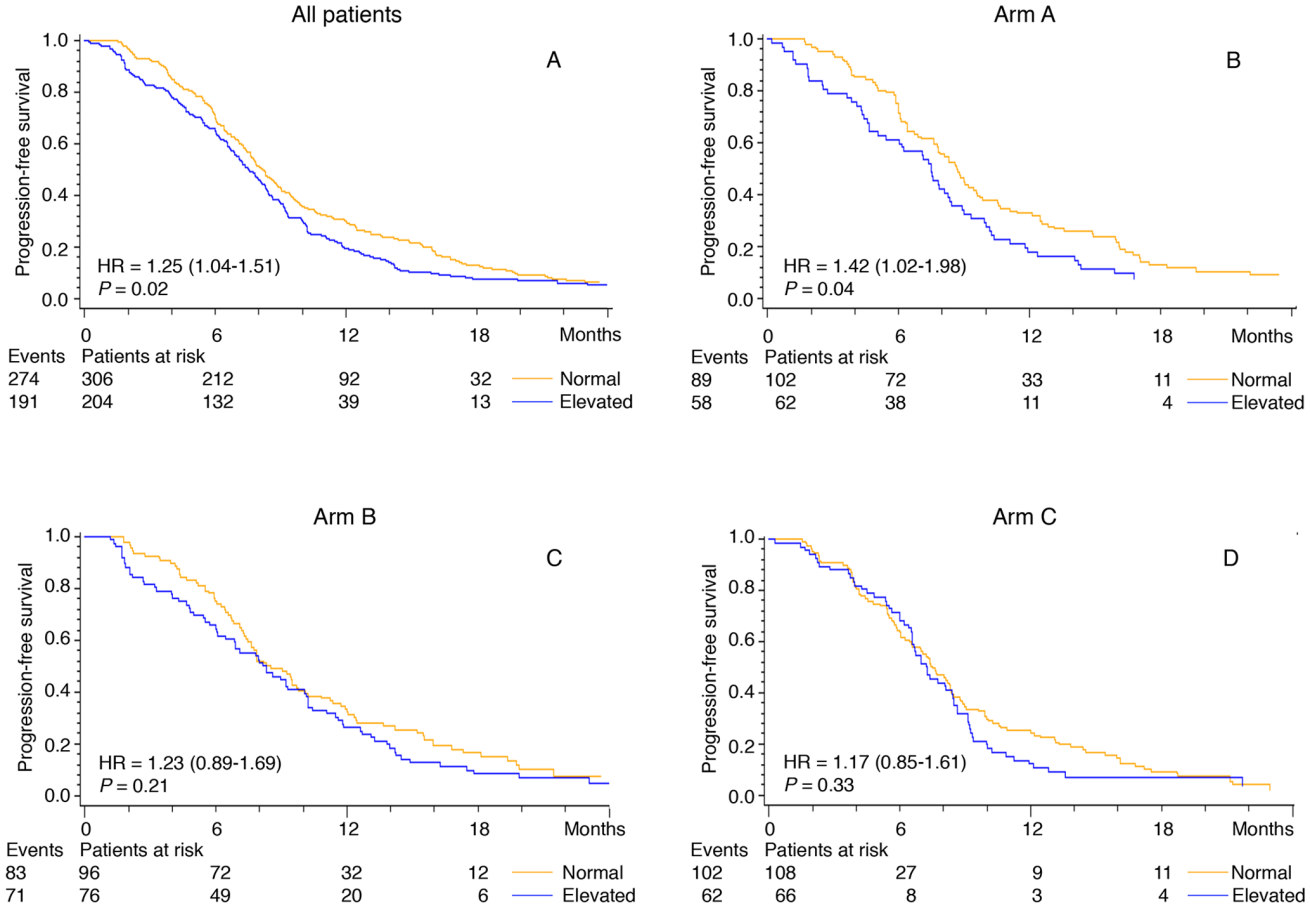
Kaplan-Meier curves showing the association between pretreatment plasma YKL-40 and PFS. For patients with metastatic colorectal cancer included in the NORDIC VII Study. All patients (1A), patients treated in arm A (1B), arm B (1C), and arm C (1D). Patients are dichotomized according to the age-corrected plasma YKL-40 (95 percentile level in healthy subjects). The P-value refers to the log-rank test for equality of strata.

**Figure 3:**
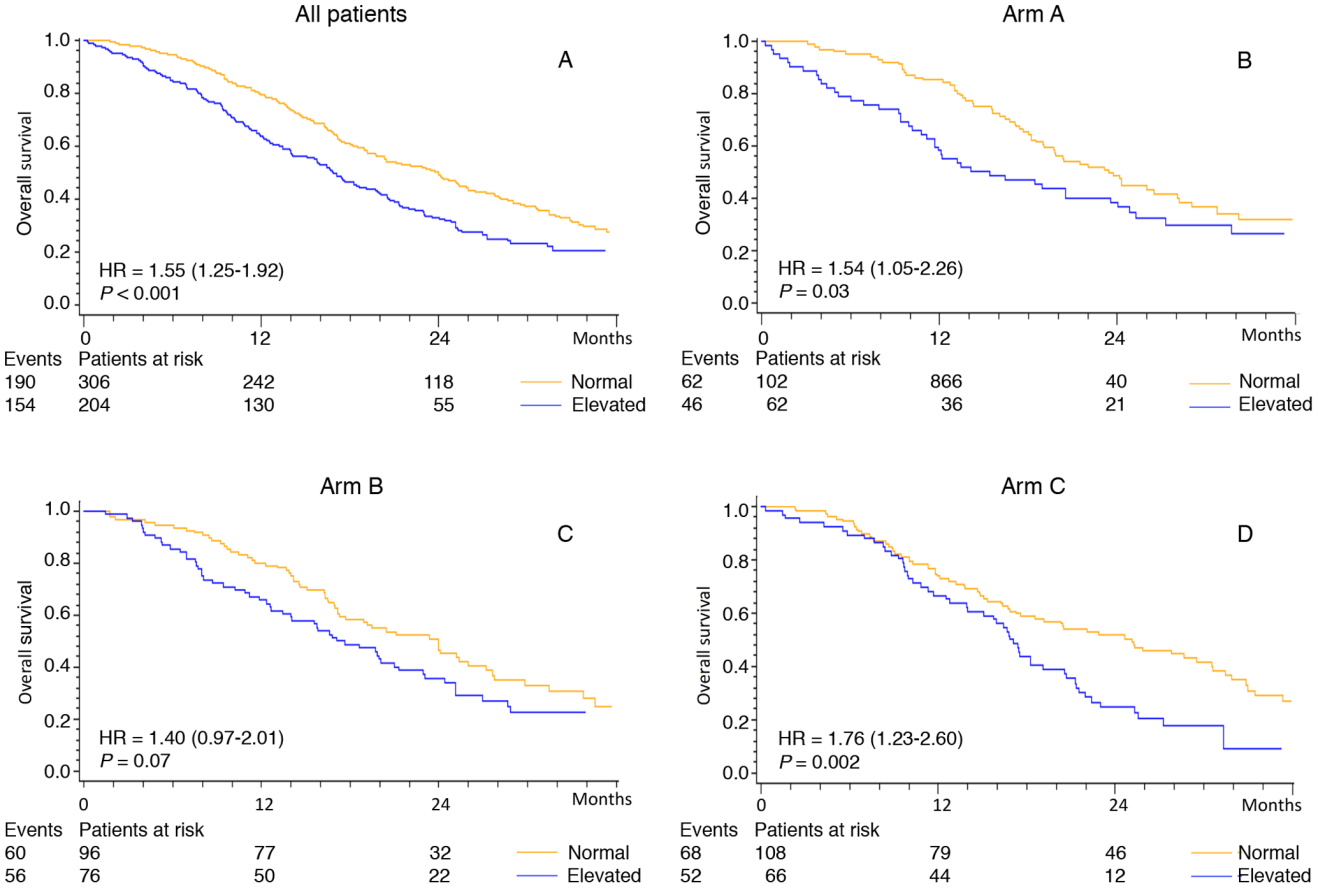
Kaplan-Meier survival curves showing the association between pretreatment plasma YKL-40 and OS. For patients with metastatic colorectal cancer included in the NORDIC VII Study. All patients (2A), patients treated in arm A (2B), arm B (2C), and arm C (2D). Patients are dichotomized according to the age-corrected plasma YKL-40 (95 percentile level in healthy subjects). The P-value refers to the log-rank test for equality of strata.

Kaplan-Meier curves revealing the association between pretreatment plasma YKL-40 and PFS and OS are shown in Figure 2 and Figure 3, respectively.

### Pretreatment plasma YKL-40 and response to therapy

While there was no statistically significant difference in pretreatment plasma YKL-40 between non-responders and responders (median 126 µg/L vs. 114 µg/L, *P* = 0.10), pretreatment plasma YKL-40 was lower in patients who became resectable during treatment (*N* = 42) compared to never resectable patients (median 93 µg/L (range 28–516) vs. 123 µg/L (range 16–3935), *P* = 0.031, Wilcoxon rank sum). Thirty-one percent of the resected patients had elevated plasma YKL-40 compared to 41% (*P* = 0.21, CHI-sq) of the never-resected patients. For comparison, there was no difference in serum CEA between patients who became resectable compared to the never-resectable patients (median CEA 36 µg/L vs. 37 µg/L, *P* = 0.88).

### Changes in plasma YKL-40 and serum CEA during treatment and efficacy

Plasma YKL-40 and serum CEA were determined in samples collected at baseline, at 1, 2, and 8 weeks after start of treatment, and end of treatment. After 8 weeks of treatment (*N* = 409) statistically significant associations of short OS with high plasma YKL-40 was found for (continuous variable; HR = 1.14; 95% CI 1.02–1.28; *P* = 0.027) and for high serum CEA (continuous variable; HR = 1.18; 95% CI 1.12–1.25; *P*<0.0001) were found. In contrast, short PFS was not associated with either high plasma YKL-40 (continuous variable; HR = 1.06; 95% CI 0.96–1.17; *P* = 0.28) or high serum CEA (HR = 1.08; 95% CI 1.03–1.13; *P* = 0.26). An increase in plasma YKL-40 during treatment evaluated by the ratio of the updated plasma YKL-40 (i.e. level after 1, 2, and 8 weeks of treatment and at end of treatment compared to the baseline level) was associated with short OS (HR = 1.27; 95% CI 1.06–1.52; *P* = 0.011), but not with short PFS (HR = 1.00; 95% CI 0.87–1.15; *P* = 0.98).

## Discussion

We assessed the prognostic value of plasma YKL-40 at baseline and during treatment of patients with mCRC included in the NORDIC VII Study. This is a phase III trial of cetuximab with continuous or intermittent oxaliplatin-5-flourouracil versus oxaliplatin-5-flourouracil alone in first-line treatment [5]. We found that 40% of the patients had an elevated pre-treatment plasma concentration of YKL-40 (i.e. above the age-corrected 95 percentile in healthy subjects). Univariate analyses showed that high pretreatment plasma YKL-40 was associated with short PFS and OS. Multivariate analysis (plasma YKL-40, *KRAS* and *BRAF* mutational status, WHO PS, number of metastatic sites, serum CEA, serum ALP, and serum CRP) showed that high plasma YKL-40 was an independent biomarker of short OS but not of short PFS. We did not find an interaction of the prognostic value of plasma YKL-40 and therapy, i.e. Nordic FLOX with or without cetuximab.

Our study supports previous findings of an association between elevated pretreatment plasma concentrations of YKL-40 and poor prognosis of patients with CRC [20]–[22]. Few studies have evaluated whether changes in plasma YKL-40 can be used to monitor treatment efficacy in patients with cancer. To our knowledge, this is the first study to investigate changes in plasma YKL-40 during treatment with first-line chemotherapy with or without cetuximab in patients with mCRC. After 8 weeks and at end of first-line treatment high plasma YKL-40 was associated with short OS, and the updated ratio of plasma YKL-40 (i.e. a increase in the concentration of plasma YKL-40 compared to baseline level) during treatment was significantly associated with short OS. These results indicate that plasma YKL-40 may be a useful biomarker of inflammation to monitor in patients with mCRC during treatment. However, these results need to be validated in future studies. Others have reported that plasma YKL-40 can be used as a biomarker for monitoring cancer recurrence and prognosis after operation for CRC [21], melanoma stages I and II [34], high grade glioblastomas [14], [35], head and neck cancer after radiotherapy [36], and for metastatic prostate cancer after hormone therapy [37].

The mechanism behind short OS in cancer patients with elevated plasma YKL-40 is not fully understood. Cancer cells with a high production of YKL-40 may have an “aggressive phenotype,” with high proliferation and differentiation rates or metastatic potential. Also, YKL-40 may play a causal role in the pathogenesis of gastrointestinal cancer, and may be a biomarker of gastrointestinal inflammation [38]. There is increasing evidence that the tumor microenvironment supports tumor development, growth, and metastatic potential [39]. An important part of this microenvironment is tumor-promoting inflammation that is seen in virtually all CRC lesions [40]. YKL-40 is produced by cancer cells (including CRC cells) and by macrophages and neutrophils in areas with inflammation surrounding cancer cells [41] (and personal observations). In *vitro* studies and tumor models in mice have demonstrated that YKL-40 plays a role in several biological processes such as inflammation; angiogenesis, both independently and through stimulating vascular endothelial growth factor; apoptosis; cell proliferation and differentiation; and regulation of cancer cell growth and metastatic potential [10], [13]–[15], [42], [43], all hallmarks of cancer as described by Hanahan et al [39]. In addition, it has been demonstrated that YKL-40 binds to the IL-13 receptor α2 with high affinity and that the YKL-40-IL-13Ra2-TGF-b1 axis play a role in the progression of malignant melanoma [19]. However, future studies are required to develop our understanding of the function of YKL-40 in cancer development and progression.

It remains to be established whether YKL-40 plays a role in the pathogenesis of CRC and other diseases characterized by inflammation and tissue remodeling, whether these diseases lead to increased plasma YKL-40, or whether inflammation, tissue remodeling, or some other factors cause both increased plasma YKL-40 and these diseases. Plasma YKL-40 is an acute-phase protein like serum CRP but in contrast to CRP YKL-40 is produced locally in areas with inflammation by cancer cells, macrophages, and leucocytes.

The strengths of our study are a relatively large sample size and the robustness of the YKL-40 protein to multiple thawing/refreezing cycles and to delays in processing of plasma samples, for up to 3 hours. Moreover, there is no circadian variability in plasma YKL-40, and the YKL-40 ELISA has a low long-term CV. These characteristics make our results reliable, and the YKL-40 analysis is attractive in the clinical setting where plasma YKL-40 may be used as a factor in risk stratification of patients with mCRC and selection of treatment. Our study had some limitations, firstly it was a retrospective biomarker exploratory study, and may suffer from multiple comparison data fitting. Secondly, the NORDIC VII Study did not meet its endpoint, i.e. it failed to show a significant benefit of adding cetuximab to NORDIC FLOX regimen in first-line treatment of patients with mCRC. It was therefore not possible to estimate whether plasma YKL-40 could be a predictive biomarker for response to cetuximab treatment.

In conclusion, plasma YKL-40 is a new, independent prognostic biomarker in patients with mCRC treated with first-line oxaliplatin-based therapy, with or without cetuximab. Furthermore, our results show that changes in plasma YKL-40 during treatment may be useful for monitoring cancer progression. The predictive value of plasma YKL-40 could not be clarified.
