# Correction: Plasma YKL-40 in Patients with Metastatic Colorectal Cancer Treated with First Line Oxaliplatin-Based Regimen with or without Cetuximab: RESULTS from the NORDIC VII Study

**DOI:** 10.1371/journal.pone.0098836

**Published:** 2014-05-22

**Authors:** The PLOS ONE Staff

There is an error in Table 2. The values for Plasma YKL-40 in the Multivariate PFS column and the Multivariate OS column are incorrect. The author has provided a corrected table here.

**Table 2:** Univariate and multivariate Cox analyses of PFS and OS in 510 patients with mCRC included in the Nordic VII Study according to pretreatment plasma YKL-40 and clinical parameters. All treatment groups are combined.

	Progression-free Survival						Overall Survival					
	Univariate Cox analyses			Multivariate Cox analyses			Univariate Cox analyses			Multivariate Cox analyses		
	HR	95% CI	*P* value	HR	95% CI	*P* value	HR	95% CI	*P* value	HR	95% CI	*P* value
Treatment arm B vs A	0.95	0.76–1.29	0.71	0.95	0.75–1.19	0.66	1.07	0.82–1.39	o.062	1.02	0.78–1.33	0.98
Treatment arm C vs A	1.22	0.98–1.53	0.08	1.27	1.01–1.59	0.039	1.09	0.84–1.41	0.51	1.02	0.79–1.33	0.88
Plasma YKL-40 (log)*	1.11	1.03–1.20	0.006	0.99	0.90–1.08	0.75	1.18	1.06–1.32	0.002	1.12	1.01–1.25	0.033
Age per 10 years	0.94	0.85–1.04	0.21	0.93	0.84–1.03	0.17	0.98	0.87–1.11	0.79	0.99	0.88–1.12	0.85
Sex, Female vs. male	1.09	0.91–1.32	0.34	0.97	0.80–1.16	0.77	0.89	0.71–1.12	0.33	0.83	0.66–1.03	0.092
*KRAS,* Mutant vs. WT	1.16	0.95–1.42	0.15	1.37	1.09–1.71	0.006	1.06	0.84–1.34	0.63	1.50	1.13–1.91	0.004
*BRAF*, Mutant vs. WT	1.99	1.45–2.72	<0.0001	2.56	1.81–3.63	<0.0001	4.55	3.03–6.84	<0.0001	4.71	3.21–6.93	<0.0001
No. of metastatic sites, >1 vs. 1	1.42	1.16–1.75	0.0007	1.40	1.14–1.72	0.001	1.51	1.19–1.92	0.0008	1.44	1.13–1.85	0.003
WHO PS, ≥1 vs. 0	1.65	1.36–2.00	<0.0001	1.40	1.13–1.73	0.002	1.98	1.59–2.46	<0.0001	1.55	1.21–1.97	0.0003
Serum CRP, Elevated vs. normal	1.52	1.25–1.84	<0.0001	1.35	1.09–1.68	0.006	1.65	1.32–2.07	<0.0001	1.31	1.03–1.69	0.030
Serum CEA, Elevated vs. normal	1.64	1.27–2.10	<0.0001	1.62	1.24–2.12	0.0005	1.90	1.40–2.58	<0.0001	1.94	1.39–2.70	<0.0001

HR  =  Hazard ratio. CI  =  Confidence interval. *Plasma YKL-40 was included as a log transformed continuous variable.

There are also several errors in the third sentence of the “Changes in plasma YKL-40 and serum CEA during treatment and efficacy” section of the Results, including an incorrect P-value for the high-serum CEA. The correct sentence is: In contrast, short PFS was not associated with high plasma YKL-40 (continuous variable; HR  =  1.06; 95% CI 0.96-1.17; P  =  0.28) but with high serum CEA (HR  =  1.08; 95% CI 1.03-1.13; P  =  0.0026).
